# Caregiver burden related to feeding process in Alzheimer’s disease

**DOI:** 10.1590/1980-5764-DN-2022-0092

**Published:** 2023-07-24

**Authors:** Verônica Salazar Moreira, Márcia Lorena Fagundes Chaves, Raphael Machado de Castilhos, Maira Rozenfeld Olchik

**Affiliations:** 1 Universidade Federal do Rio Grande do Sul, Programa de Pós-Graduação em Medicina, Ciências Médicas, Porto Alegre RS, Brazil.; 2 Universidade Federal do Rio Grande do Sul, Faculdade de Medicina, Departamento de Medicina Interna, Porto Alegre RS, Brazil.; 3 Hospital de Clínicas de Porto Alegre, Serviço de Neurologia, Porto Alegre RS, Brazil.; 4 Universidade Federal do Rio Grande do Sul, Faculdade de Odontologia, Curso de Fonoaudiologia, Departamento de Cirurgia e Ortopedia, Porto Alegre RS, Brazil.

**Keywords:** Alzheimer Disease, Deglutition Disorders, Caregiver Burden, Feeding Behavior, Doença de Alzheimer, Transtornos de Deglutição, Fardo do Cuidador, Comportamento Alimentar

## Abstract

Difficulties in the feeding process, such as aversive feeding behaviors and dysphagia, are common in patients with Alzheimer’s disease (AD) and can often overload their caregivers. Although dysphagia is already established as a factor contributing to caregiver burden, the impact of aversive behaviors is less studied.

**Objectives:**

Evaluate the relationship between the feeding process in individuals with AD and their caregiver’s burden.

**Methods:**

Dyads of individuals with AD and their caregivers were recruited for a cross-sectional study. The Edinburgh Feeding Evaluation in Dementia (EdFED) scale, the Zarit Burden Interview (ZBI), the mini-mental state examination (MMSE), the Functional Activities Questionnaire (FAQ), and the Functional Oral Intake scale (FOIS) were performed.

**Results:**

We included 60 AD individuals-caregivers dyads. The median (IQR) age of caregivers was 57 (19–81) years, and the most were females (70%). The individuals with AD had a median MMSE of 12 (6–15), and the disease duration was 4 (2–6) years. The mean (SD) Zarit score was 20.95 (6.51). In the multivariate linear regression, the EdFED score (95% CI 0.368–1.465) and time as a caregiver (95% CI 0.133–1.355) were associated with the caregiver’s burden.

**Conclusions:**

Aversive behaviors were associated with the caregiver burden of individuals with AD, even with a short duration of the disease. These findings show the importance of education for caregivers regarding the feeding process, as these measures have great potential to minimize the caregiver’s burden.

## RESUMO

Dificuldades no processo de alimentação, como comportamentos alimentares aversivos e disfagia, são comuns em indivíduos com doença de Alzheimer (DA) e muitas vezes podem sobrecarregar seus cuidadores. Embora a disfagia já esteja estabelecida como um fator que contribui para a sobrecarga do cuidador, o impacto dos comportamentos aversivos é menos estudado.

**Objetivos:**

Avaliar a relação entre o processo de alimentação em indivíduos com DA e a sobrecarga de seus cuidadores.

**Métodos:**

Díades de indivíduos com DA e seus cuidadores foram recrutados para um estudo transversal. Os protocolos *Edinburgh Feeding Evaluation in Dementia Scale* (EdFED), *Zarit Burden Interview* (ZBI), *Mini-Mental State Examination* (MMSE), *Functional Activities Questionnaire* (FAQ) e *Functional Oral Intake Scale* (FOIS) foram realizados.

**Resultados:**

Incluímos 60 díades de cuidadores-indivíduos com DA. A idade mediana (intervalo interquartil — IQR) dos cuidadores foi de 57 (19–81) anos e a maioria era do sexo feminino (70%). A mediana do MMSE dos indivíduos com DA foi de 12 (6–15) e o tempo de doença foi de quatro (2–6) anos. A pontuação média do Zarit foi de 20,95 (6,51). Na regressão logística multivariada, o escore EdFED (intervalo de confiança de 95% — IC95% 0,368–1,465) e o tempo como cuidador (IC95% 0,133–1,355) foram associados à sobrecarga deste.

**Conclusões:**

Comportamentos aversivos foram associados à sobrecarga do cuidador de indivíduos com DA, mesmo com pouco tempo de doença. Esses achados mostram a importância da educação dos cuidadores quanto ao processo de alimentação, pois essas medidas têm grande potencial para minimizar sua sobrecarga.

## INTRODUCTION

A pproximately 50 million people worldwide are affected by some form of dementia, a number estimated to triple by 2050, with two-thirds coming from low- and middle-income countries. Alzheimer’s disease (AD) is the leading cause of dementia and arguably one of the most debilitating and expensive diseases^1,2^. AD has a progressive and unrelenting course, causing a loss of ability to perform routine tasks and the need for supervision and care from third parties^3,4^.

With the progression of the disease, feeding difficulties may arise, especially aversive feeding behaviors (ABFs)^5–8^ and interruption of necessary preparatory actions for swallowing^9^. AFBs hinder or prevent oral nutrition due to various changes, such as food refusal, resistance, and dysphagia, presented by patients^6^. Dysphagia is a common manifestation in moderate to severe stages, and its frequency ranges from 84% to 93% of patients^10–13^. Several complications secondary to dysphagia can occur, such as aspiration pneumonia, dehydration, and malnutrition, which ultimately can lead to the patient’s death^14^.

As the disease progresses, care and supervision are necessary, as difficulties in managing activities of daily living also arise^4^. This care is usually offered by a family member without remuneration. Caring for individuals with AD produces psychological, emotional, and financial distress for their caregivers due to the gradual loss of cognitive functions that can evolve into total dependence, causing a burden on the family nucleus and especially on the main caregiver.

The so-called caregiver burden (CB) is a very frequent condition and can lead to a reduction in the caregiver’s quality of life and a worsening of the patient’s behavioral symptoms. Several factors associated with CB are known, related to the patient as disease severity and neuropsychiatric manifestations, and to the caregiver as time of caregiving and their physical and mental health^15^. In addition to these known factors, problems related to the feeding process, such as preparing and offering meals, nutritional support, and weight loss, can also add complexity to patient care, especially in the later stages of dementia^16^.

A previous study on this topic showed that initial feeding difficulties were significantly associated with the caregiver’s age, the disease’s severity, and the initial patient’s autonomy and psychological functioning. A logistic regression analysis also showed a positive association between AFBs worsening and the initial caregiver’s burden after controlling for confounding factors. This study concluded that both cognitive impairment and family stress can help in predicting which individuals with AD living at home will develop AFBs. They inferred that nutritional information and support for families are probably the best strategies to prevent AFBs during AD and to improve consequently the patient’s and caregiver’s quality of life^17^.

Other studies showed on multivariate analyses that decline in feeding were significantly associated with increased caregiver burden^18^ and that patients with dementia needed supervision (50%), as well as physical help during mealtime (40%). The caregiver burden score was positively correlated with the EdFED-Q score (r=0.405, p<0.05). Furthermore, multiple regression analysis showed that after adjustment for age, the EdFED-Q score remained correlated with caregiver burden. They concluded that caregiver burden is associated with feeding problems and functional disability among patients with dementia, and there is a need to educate the caregivers to improve the quality of life of both the carers and the demented patients^19^.

In this context, although dysphagia is already established as a predictor of CB, few studies have focused on the role of aversive behaviors and other changes related to feeding in this process. Thus, we aim to identify the impact of the feeding process on the burden of caregivers of patients with AD.

## METHODS

### Participants

Dyads of individuals with AD and their carers were recruited, according to McKhann et al.^20^ criteria, from the dementia outpatient clinic in a reference hospital in southern Brazil in the period from June to November 2021. We included non-institutionalized patients only, primary caregivers, family, and unpaid workers older than 18 years. Caregivers who were not family members or were paid were excluded, as well as the patients who have other associated neurological diseases.

All caregivers signed a free and informed consent form, and the project was approved by the local Research Ethics Committee (GPPG 2021–0004).

For the sample size calculation, we used the PSS Health tool online, version^21^. We considered a power of 80%, a significance level of 5%, and a standard deviation of 5.0531 points, with a total sample size of 56 subjects.

### Instruments

Data collection was performed through instruments before the routine consultation or over the phone. The instruments used by a trained speech therapist.

Approximately 70% of the data collection was done over the phone. Although most of the instruments are not validated for this application, as we are in a pandemic period and the questions are multiple choice, with the possibility for the applicator to read the questions, it is inferred that there was no loss in the results.

The patient’s medical record was consulted to collect information regarding sociodemographic variables, disease staging, and previously used neurological scales, specifically the mini-mental state examination (MMSE)^22^ and the Functional Activities Questionnaire (FAQ)^23^. The MMSE is a cognitive function assessment based on a possible score of 30 points. Higher score values indicate higher cognitive performance^22^. The FAQ is a scale applied by the caregiver to discuss the patient’s ability to perform certain functions. The score ranges from 0 to 30, and the lower the score obtained, the greater their independence and autonomy^23^.

During the caregiver interview, we collected the following data:

- Sociodemographic variables include age, sex, kinship, years of formal education, family income, and other questions about instructions on the feeding process and speech therapy.
- Functional Oral Intake scale (FOIS)^24^ is a 7-point ordinal scale describing the functional level of oral intake of food and liquids. Level 7 represents a total oral diet with no restrictions, levels 6–4 indicate a total oral diet with restrictions, levels 3–2 describe a mixed oral and tube intake, and level 1 represents a tube-dependent intake.
- The Edinburgh Feeding Evaluation in Dementia (EdFED) scale^25^ is a 10-item questionnaire that assesses the eating behavior of patients with dementia. The first two items refer to the need for assistance during meals. Items 3 and 4 are indicators of the person’s difficulty in eating. Items 5–10 describe feeding behaviors. The score ranges from 0 to 20, with higher scores meaning higher modifications in feeding behavior.
- Zarit Burden Interview (ZBI) – Short Version^26^ consists of 12 questions assessing the impact of a burden on the following aspects of the caregiver’s life: health, social and personal life, financial and emotional well-being, and interpersonal relationships. The answers for each question comprise a scale from 0 to 4, in which 0=never, 1=rarely, 2=sometimes, 3=often, and 4=always. An overall score of 0–48 is obtained, with a higher score meaning a greater burden perception.

### Data analysis

Continuous variables were described as mean (standard deviation) or median (interquartile range, IQR) according to their distribution, and categorical variables as frequency and percentage. We performed linear regression analysis using the ZBI as the dependable variable and patient age, disease duration, time as a caregiver, levels of education, MMSE, FOIS, and EdFED as independent variables. The analysis was performed using the IBM SPSS version 18 for Windows.

## RESULTS

We included 60 AD individuals-caregivers dyads. Their characteristics are summarized in Table 1.

**Table 1:** Characteristics of Alzheimer’s disease individuals and their caregivers.

Characteristics	Patients	Caregivers
Age, median (IQR)	78 (70–83)	57 (45–67)
Sex (female), *n* (%)	36 (60)	42 (70)
Kinship (son/daughter), *n* (%)	–	40 (67)
Education (years), median (IQR)	5 (3–7.5)	8 (6–12)
Marital status (married), %	55	75
Disease duration (years), median (IQR)	4 (2–6)	–
Time as a caregiver (years), median (IQR)	–	5 (3–5)
FAQ, median (IQR)	26 (18–30)	–
MMSE, median (IQR)	12 (6–15)	–
FOIS	7 (5–7)	–
EDFED, median (IQR)	3 (1.5–6)	–
Zarit, mean (SD)	–	20.95 (6.5)

Abbreviations: IQR: interquartile range; FAQ: Functional Activities Questionnaire; MMSE: mini-mental state examination; FOIS: Functional Oral Intake scale; EDFED: The Edinburgh Feeding Evaluation in Dementia scale; Zarit: Zarit Caregiver Burden scale; SD: standard deviation.

On FOIS, most patients had no restrictions on oral feeding (66.6%, n=40). 28.3% (n=17) had total oral feeding with multiple consistencies but with the need for special preparation or compensations. FOIS correlated moderately with time as a caregiver (Rho=–0.405; p=0.001) and had a weak correlation with patient age (Rho=-0.394; p=0.002) and the Zarit Burden Interview (Table 2). Here, it is worth noting that time as a caregiver means how long the caregiver is caring for the patient included in the study.

**Table 2:** Functional Oral Intake scale from Alzheimer’s disease individuals.

	Item	% (n)
1	No oral intake	0
2	Tube dependent with minimal/inconsistent oral intake	1.7 (1)
3	Tube supplements with consistent oral intake	0
4	Total oral intake of a single consistency	3.3 (2)
5	Total oral intake of multiple consistencies requiring special preparation	28.3 (17)
6	Total oral intake with no special preparation, but must avoid specific food or liquid items	0
7	Total oral intake with no restrictions	66.7 (40)

Regarding eating behaviors, evaluated by EdFED, the median (IQR) was 3 (1.5–6). Most patients need constant supervision (43.3%, n=26) or physical help (35%, n=21) during meals. These and other findings from the questionnaire are shown in Table 3. However, only 10% (n=6) of the caregivers declared they had been instructed on the feeding process and its possible consequences, and only 5% (n=3) of the patients had been previously evaluated by a speech therapist.

**Table 3:** The Edinburgh Feeding Evaluation in Dementia scale item distribution.

	Item	0=never happens, % (n)	1=happens sometimes, % (n)	2=happens often, % (n)
1	Does the patient require close supervision while feeding?	13.3 (8)	43.3 (26)	43.3 (26)
2	Does the patient require physical help with feeding?	33.3 (20)	35 (21)	31.7 (19)
3	Is there spillage while feeding?	50 (30)	43.3 (26)	6.7 (4)
4	Does the patient tend to leave food on the plate at the end of a meal?	51.6 (31)	41.6 (25)	6.6 (4)
5	Does the patient ever refuse to eat?	83.3 (50)	15 (9)	1.7 (1)
6	Does the patient turn his/her head away while being fed?	80 (48)	20 (12)	0
7	Does the patient ever refuse to open his/her mouth?	83.3 (50)	16.7 (10)	0
8	Does the patient spit out his/her food?	93.3 (56)	6.7 (4)	0
9	Does the patient leave his/her mouth open allowing food to drop out?	88.3 (53)	11.7 (7)	0
10	Does the patient refuse to swallow?	95 (57)	5 (3)	0

**Figure 1:**
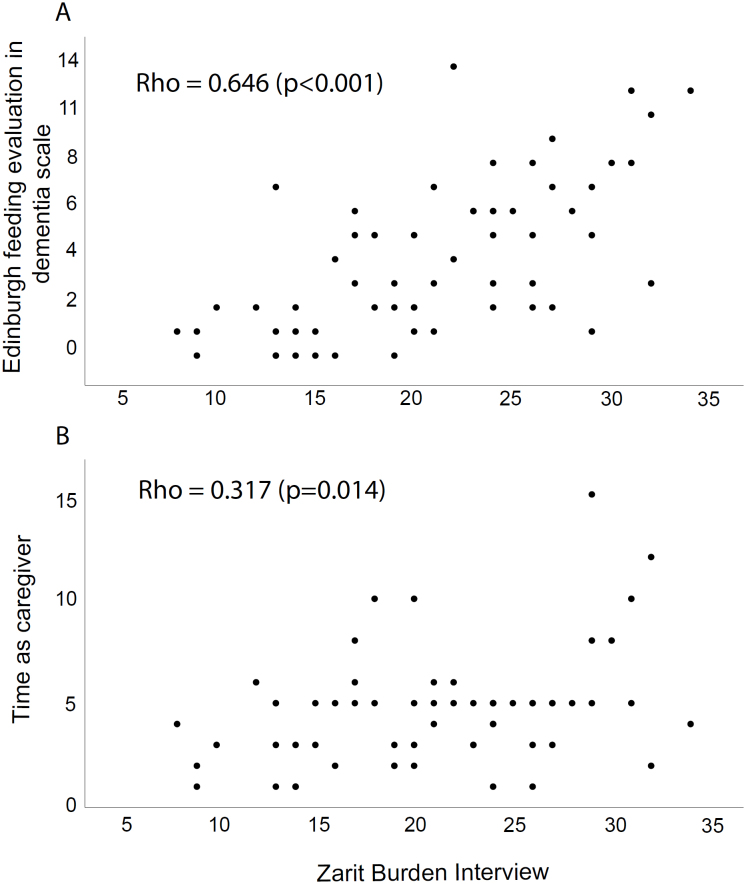
Correlation of Zarit Burden Interview with Edinburgh Feeding Evaluation in Dementia scale (A) and time as caregiver (B).

The mean (SD) Zarit caregiver burden score was 20.95 (6.5). Zarit score correlated moderately with EdFED (Rho=0.646; p<0.001) and weakly with patient age, disease duration, time as a caregiver, diet consistency, and cognitive status (Table 4). In the linear regression using these variables as independent ones and Zarit as the outcome, only EdFED (standard coefficient 0.572; p<0.001) and time as a caregiver (0.232; p=0.049) were significant (Table 4 and Figure 1).

**Table 4:** Linear regression using Zarit Burden Interview as the dependent variable and its correlation with independent variables.

Variables	Correlation with ZBI		Linear regression	
	Rho	p-value	Standardized coefficient (95% CI)	p-value
Patient age	0.279	0.031	0.133 (-0.061 to 0.267)	0.214
Disease duration	0.226	0.083	-0.073 (-0.775 to 0.407)	0.535
Time as caregiver	0.317	0.014	0.232 (-0.002 to 1.099)	0.049
MMSE	-0.379	0.003	-0.173 (-0.440 to 0.054)	0.122
FOIS	-0.292	0.024	0.181 (-0.428 to 2.486)	0.162
EdFED	0.646	<0.001	0.596 (0.625 to 1.658)	<0.001

Abbreviations: ZBI: Zarit Burden Interview; MMSE: mini-mental state examination; FOIS: Functional Oral Intake scale; EdFED: The Edinburgh Feeding Evaluation in Dementia scale; CI: confidence interval.

## DISCUSSION

Changes in the feeding behavior of patients with AD are predictors of caregiver burden, even if we control for patient age, disease duration, time as a caregiver, cognitive status, and diet consistency. Previous studies had already found a moderate burden on caregivers of patients who had changes in feeding behaviors^17,19,27^. The association with the EdFED scale in our study seems to be mostly determined by the items corresponding to the caregiver’s need to supervise or help the patients during feeding. The caregiver burden in our study was not related to difficult behaviors during meals, as a minority of caregivers reported these behaviors. This finding may be related to the fact that most patients have been followed up in our clinic for some time, with most difficult behaviors having already been managed, pharmacologically or not. In this way, we could understand that the simple act of supervising meals already leads to an increase in caregiver burden. Another hypothesis is that these patients, despite not referring to dietary changes, have already developed mild dysphagia, which may have already influenced the caregiver burden^27–29^.

Although changes in diet consistency were not statistically significant in the linear regression model, they correlated with caregiver burden. A proportion of patients needed some type of change in diet (31.6%), with just one patient dependent on tube feeding. This finding may be related to the need for more time to prepare modified meals, such as blending, crushing, kneading, or sieving the food after preparation^30,31^.

Our sample consisted predominantly of patients who, despite having a median disease duration of 4 years, had more severe cognitive decline, with a median MMSE of 12 (6–15). Our university hospital clinic receives patients with more severe diseases, caused by the slowness of our health system and the known difficulties of primary care physicians in identifying these patients. We found a correlation between cognitive status, eating behavior, and dietary changes. This can happen because feeding problems occur more frequently in severely ill patients^32^.

Despite the short period of illness in the sample (4 (2–6) years), there was also a correlation between burden and time as a caregiver (95%CI 0.133–1.355), which is possibly due to overload factors already expected from the caregiver, such as changes in daily routine, social isolation, anguish, fear, financial expenses, and decreased quality of life^33–35^, associated with the stress of aversive eating behaviors in AD patients.

In our study, the caregivers presented considerable burden levels, even with a short period of disease and with few changes in the patient’s diet. These findings indicate that with the progression of the disease, these levels may increase, taking into account the possibility of more difficulties in the feeding process, aggravation of aversive eating behaviors, the need to change consistency, and dysphagia.

Most studies on the feeding process and caregiver burden described in the literature were developed in countries. However, even with different realities, our main findings are similar to those found in the literature: aversive feeding behaviors, even if not so severe in our study, changes in diet, time of disease, and time as a caregiver are the main factors correlated with caregiver burden^16–18^.

Therefore, our study reinforces the need for early interventions to efficiently prevent problems arising from the eating process and, thus, reduce the level of burden and improve the quality of life of patients and caregivers. The main clinical implication of the study was to understand the importance of educational measures for the caregiver before the onset of mild changes in the swallowing process and aversive feeding behaviors, as these measures have great potential to minimize the caregiver’s burden related to the feeding process^16,17^.

In conclusion, caregivers of individuals with Alzheimer’s disease showed a considerable level of burden in our study, even with a short time of disease and with few changes in the diet. These findings indicate that with the progression of the disease, the burden levels may increase even more, considering the possibility of more difficulties in the feeding process with the worsening of aversive feeding behaviors and dysphagia.

Correct orientation of the feeding process can facilitate care and has the potential to reduce caregiver burden in this regard. Therefore, our study reinforces the need for early interventions to efficiently prevent problems arising from the feeding process and, thus, reduce the level of burden, improving the quality of life of individuals with AD and their caregivers. A global action plan of the World Health Organization is a great example and aims to improve the lives of individuals with dementia, their caregivers, and their families while decreasing the impact of dementia on communities and countries. It provides a set of actions to realize the vision of a world in which dementia is prevented and people with dementia and their caregivers receive the care and support they need to live a life with meaning and dignity^35^.

Likewise, these findings show us the importance of investing in public policies aimed at the caregiver, as it is known that the burden is inevitable in the progression of the disease and that adequate support can contribute to their well-being throughout the course of the disease.
